# Data on ecological associations and stand structure of chilgoza pine (*Pinus gerardiana* Wall. ex D. Don) in Afghanistan

**DOI:** 10.1016/j.dib.2018.03.118

**Published:** 2018-03-30

**Authors:** Mohammad Nasir Shalizi, John W. Groninger, Safiullah Khurram, Charles M. Ruffner, Owen T. Burney

**Affiliations:** a Department of Forestry and Natural Resources, Faculty of Agriculture, Kabul University, Jamal Mina, D-1006 Kabul, Afghanistan; b Department of Forestry and Environmental Resources, North Carolina State University, 2820 Faucette Dr., Campus Box 8001, Raleigh, NC 27695, United States; c Department of Forestry, Southern Illinois University, 1205 Lincoln Drive, Mail Code, 4411, Carbondale, IL 62901, United States; d John T. Harrington Forestry Research Center, New Mexico State University, Mora, NM 87732, United States

## Abstract

Reported here are original data related to the article “Indigenous knowledge and stand characteristics of a threatened tree species in a highly insecure area: Chilgoza pine in Afghanistan” (Shalizi et al., 2018) [1]. A dendrochronological summary of all known chilgoza pine tree growth increment cores collected in Afghanistan is presented in this data in brief article. Chilgoza pine trees and regeneration density profiles are reported for four provinces of eastern Afghanistan. In addition, images depicting chilgoza pine forest structure, stand conditions, and utilization impacts are presented.

**Specifications Table**

**Table:** 

Subject area	Forest ecology
More specific subject area	Stand density, dendrochronology, silvics
Type of data	Images, figures, Excel files
How data were acquired	Field Survey. Tree cores were obtained with an increment borer. Diameter at breast height was measured with a diameter tape. Images were taken with a camera or mobile phone.
Data format	Data are presented as figures, images, and raw data files.
Experimental factors	N/A
Experimental features	N/A
Data source location	Paktia, Paktika, Khost, and Laghman provinces of Afghanistan.
Data accessibility	The data are available with this article.

**Value of the data**

• The data provide first-ever scientific information on an infrequently studied and economically important tree species from an insecure region of the world.
• These data can be used as a foundation for future studies helpful toward sustaining the chilgoza pine resource in Afghanistan.
• These data offer the basis for the first published description of chilgoza pine stand dynamics in Afghanistan.

## 1 Data

**Fig. 1:**
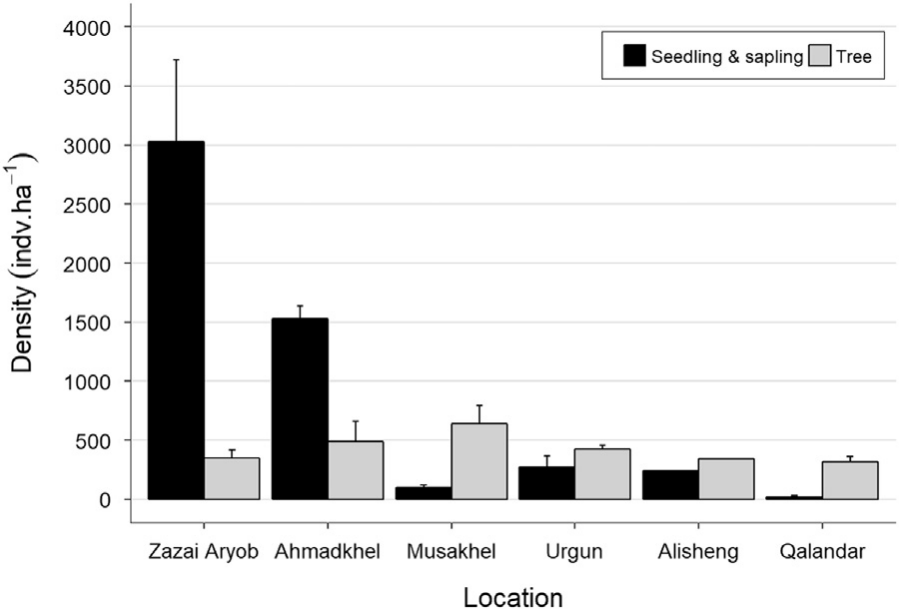
Chilgoza pine tree and seedling/sapling density (individual per hectare) at six different locations (districts) in Eastern Forest Complex of Afghanistan. Note seedling/sapling density is much higher in Zazai Aryob and Ahmadkhel districts of Paktia (details: Shalizi et al. [1]).

**Fig. 2:**
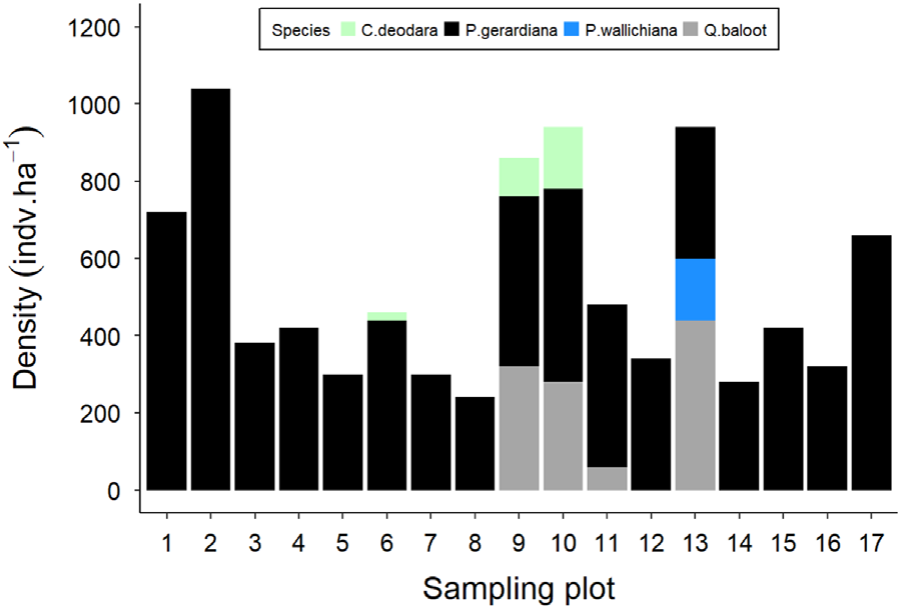
Stacked bar plot of tree species density (individuals per hectare) measured at each sampling plot. Plots 1–8 were in Khost, 9–12 in Paktika, 13 in Laghman and 14–17 were in Paktia provinces. Each color represents a different tree species.

**Fig. 3:**
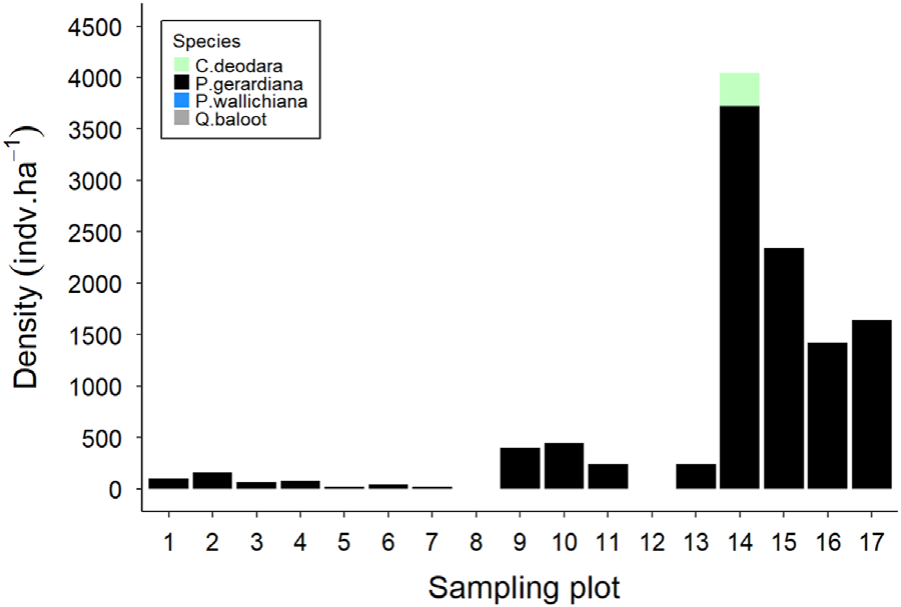
Stacked bar plot of seedling/sapling density (individuals per hectare) measured at each sampling plot. Plots 1–8 were in Khost, 9–12 in Paktika, 13 in Laghman and 14–17 were in Paktia provinces. Each color represents a different tree species.

**Fig. 4:**
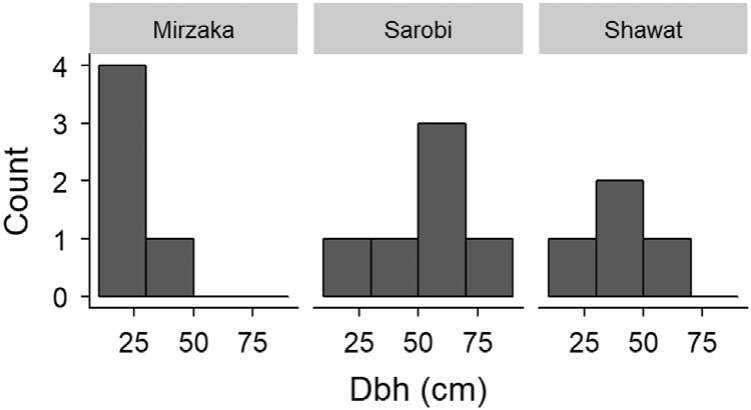
Diameter at breast height distribution of chilgoza pine measured at three different locations.

**Fig. 5:**
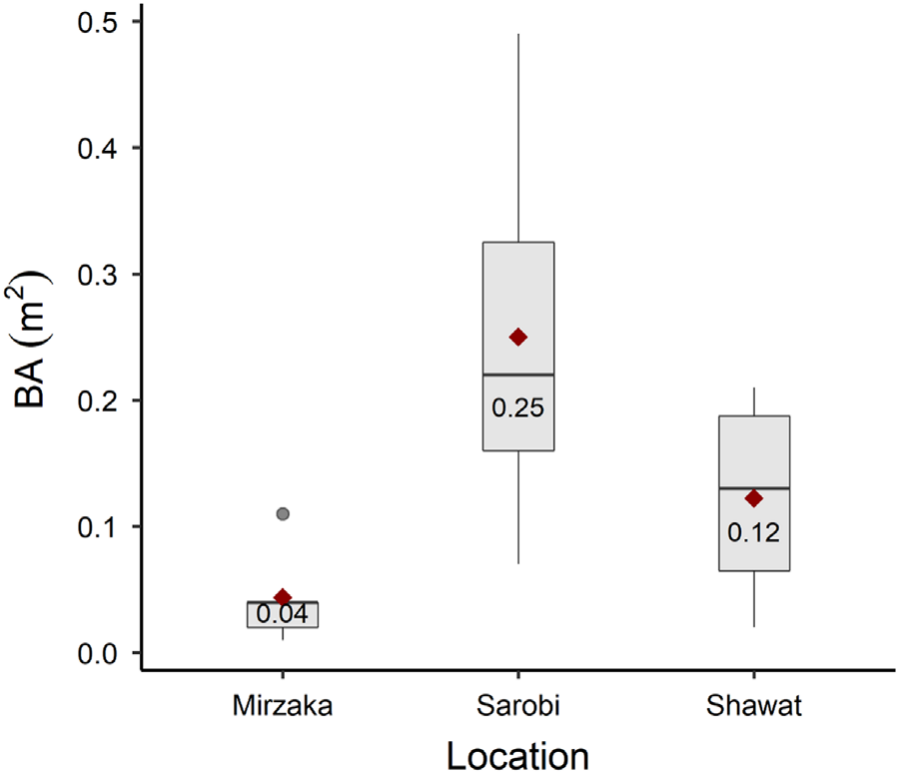
Boxplot of chilgoza pine basal area (m^2^) measured at three different locations. The red diamond is the location of the mean with its value given below it.

**Fig. 6:**
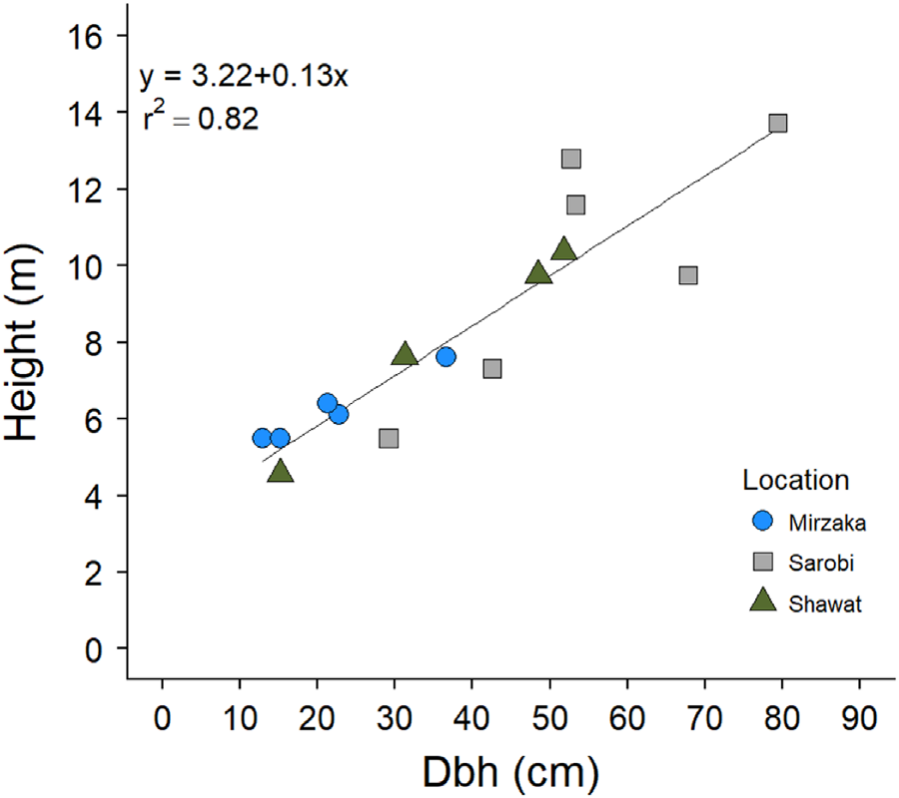
Scatterplot of the relationship between chilgoza pine height (m) and dbh (cm) measured at three different locations in Paktia and Paktika provinces of Afghanistan.

**Fig. 7:**
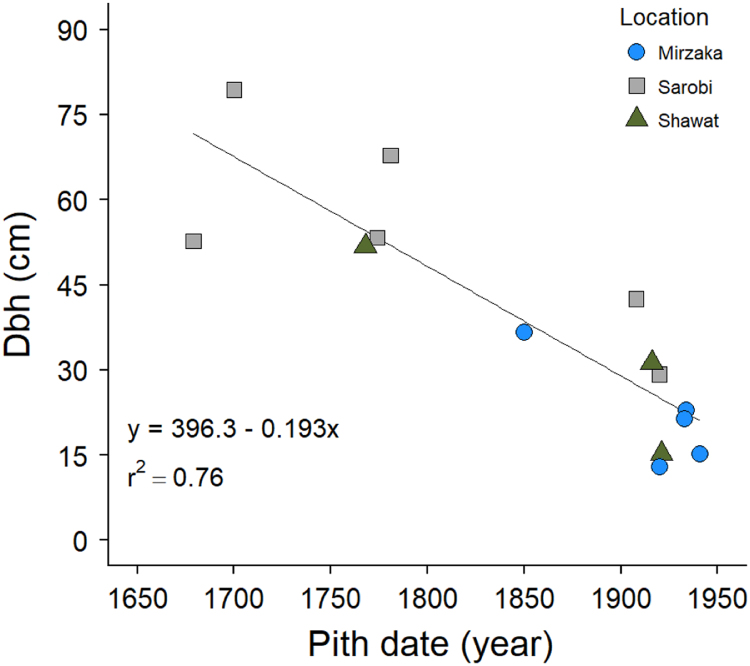
Scatterplot of relationship between chilgoza pine pith date (year) and dbh (cm) measured at three different locations in Paktia and Paktika provinces of Afghanistan.

The data in this article are divided into two parts. **Part one** provides quantitative data collected from site measurements representing 17 sampling plots across six districts in four provinces in the Eastern Forest Complex of Afghanistan. These data consist of stand density (tree and seedling/saplings per hectare) and tree growth increment core data (height, diameter at breast height (dbh), and pith date). A summary of chilgoza pine tree and natural regeneration density is presented in Fig. 1. In addition, tree and seedling/sapling density at each sampling plot is represented in Fig. 2, Fig. 3. Raw data of stand density can be found in Excel file 1. Chilgoza dbh histograms and basal area boxplot are presented in Fig. 4, Fig. 5. Scatterplots of the relationship between dbh, height, and pith date are represented in Fig. 6, Fig. 7. The raw data for these figures are provided in Excel file 2. **Part two** provide images of chilgoza forest types, examples of wood removal from chilgoza trees, and the occasion of data collection by Afghan and international scientists.

## 2 Experimental design, materials and methods

**Stand density and natural regeneration data** were collected from 17 fixed-area circular plots (radius of 12.6 m = 500 m^2^). The plots were located at six districts (4 in Musakhel, 4 in Qalandar, 4 in Urgun, 2 in Zazai Aryob, 2 in Ahmadkhel, and 1 in Alisheng) of four provinces (Khost, Paktika, Paktia, and Laghman) in the eastern forest complex of Afghanistan. Within each district, the sampling plots were established randomly, 1–5 km apart. Within each plot, dbh (diameter at 1.37 m) of the largest chilgoza tree in each plot was measured with a diameter tape. Additionally, the total number of trees by species were recorded based on two size categories; tree (> 5 cm dbh) and seedling/sapling (≤ 5 cm, ground line diameter).

**Tree cores, dbh, and height data** for live dominant trees were collected at one site each in Paktia (Shawat, Sayid Karam and Mirzaka) in October, 2009 and Paktika (Sarobi) in August, 2009 using a tree increment borer, diameter tape, and clinometer, respectively. Some tree heights were measured using photogrammetric image analysis if field time was insufficient for clinometer measurements. Increment cores were processed in the Forest History Laboratory at Southern Illinois University using standard dendroecological methods including air drying, mounting, sanding, and cross-dating cores [2]. Pith dates were determined using standard skeleton plots and visual cross-dating methods [2]. **Images** were taken by field surveyors with a camera or mobile phone camera.

## 3 Site measurement data

### 3.1 Stand density data

Tree and seedling/sapling density data of chilgoza pine were summarized for six districts and are presented in Fig. 1. Trees and seedling/sapling density data for major tree species across 17 measurement plots are presented in Fig. 2, Fig. 3.

### 3.2 Tree core data

Dbh distribution of chilgoza is presented as histograms in Fig. 4 and basal area is presented in Fig. 5. Scatterplots of height vs. dbh relationship and pith date vs. dbh relationship are given in Fig. 6, Fig. 7 respectively.

## 4 Images

**Fig. 8:**
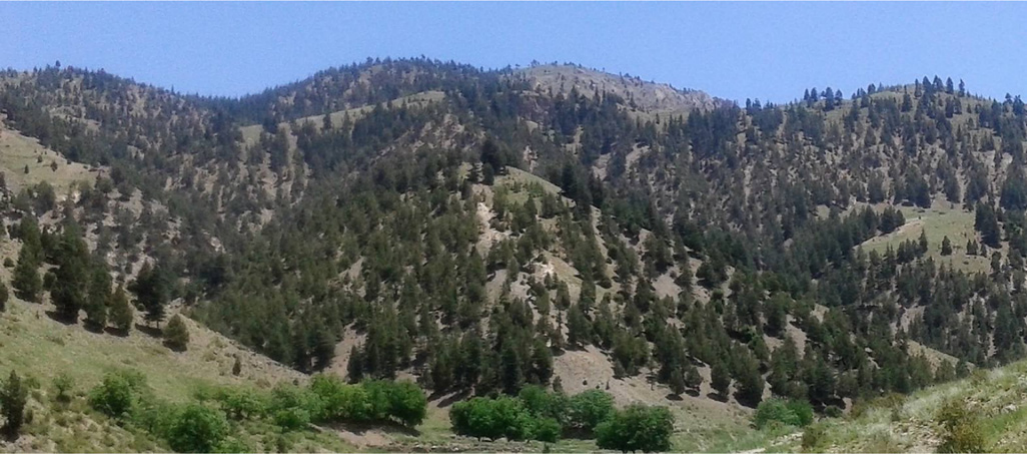
Chilgoza pine-dominated forest exhibiting high stand densities typical of a high precipitation regime (Zazai Aryob, Paktia).

**Fig. 9:**
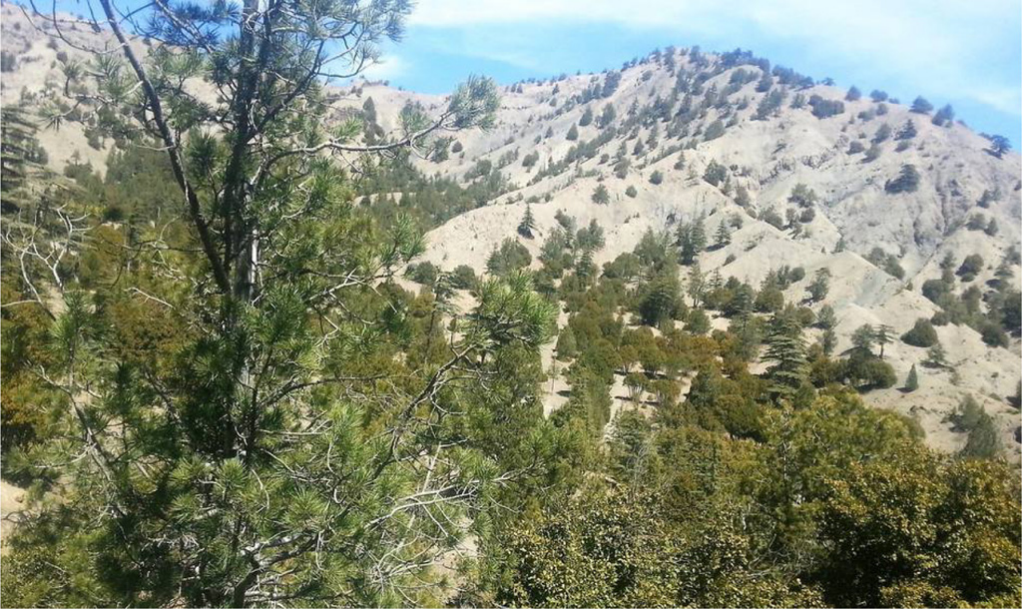
Mixed chilgoza-deodar cedar forest in Urgun district, Paktika province. Note the size of gaps between trees is much larger than the wetter site in Fig. 8.

**Fig. 10:**
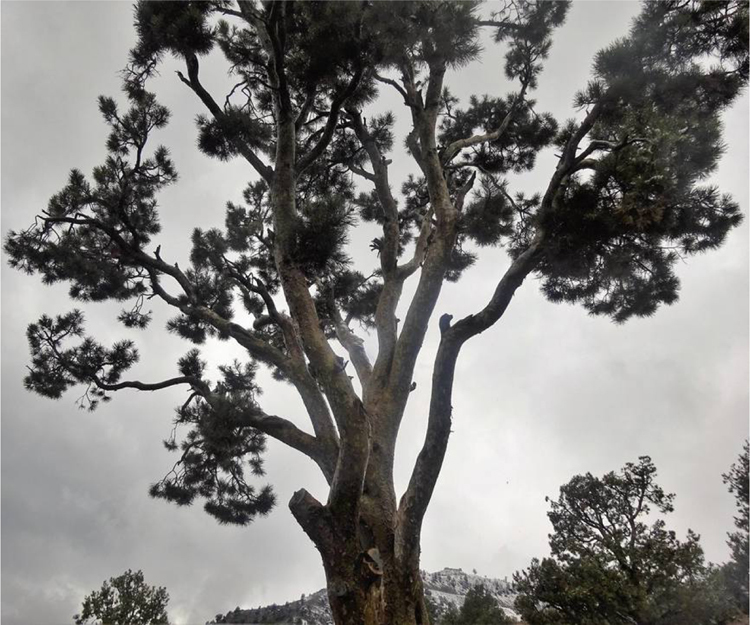
Chilgoza pine tree subjected to firewood collection and repeated cone collection (Paktia province).

**Fig. 11:**
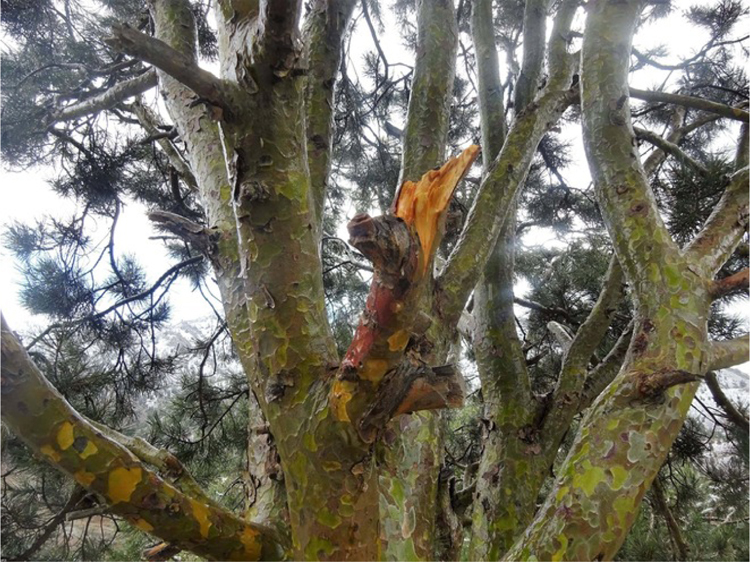
Branch of chilgoza pine tree damaged by intensive cone collection (Narai Pass, Khost-Paktia border).

**Fig. 12:**
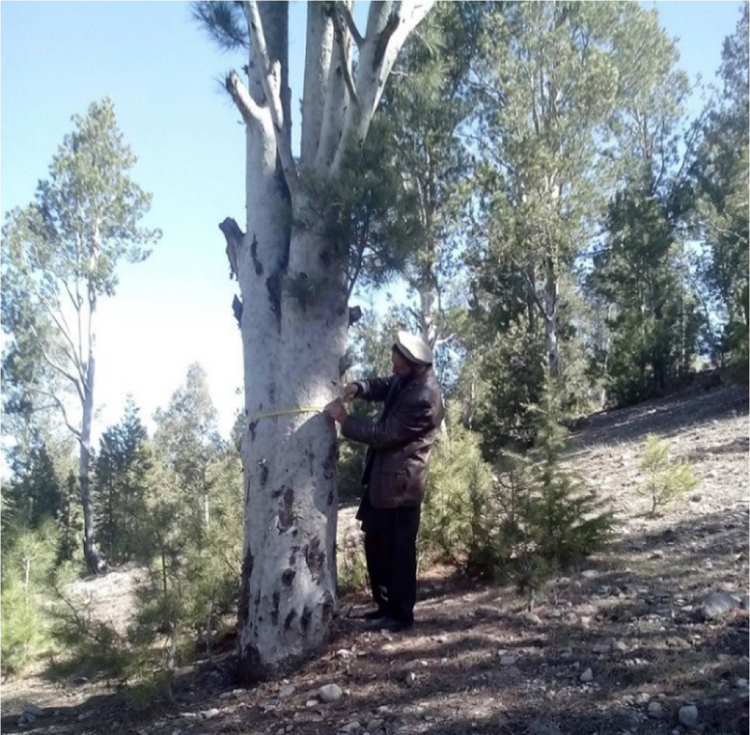
Afghan field surveyor measuring dbh of a mature chilgoza pine tree. Lower branches of this tree were removed for firewood (Zazai Aryob, Paktia province).

**Fig. 13:**
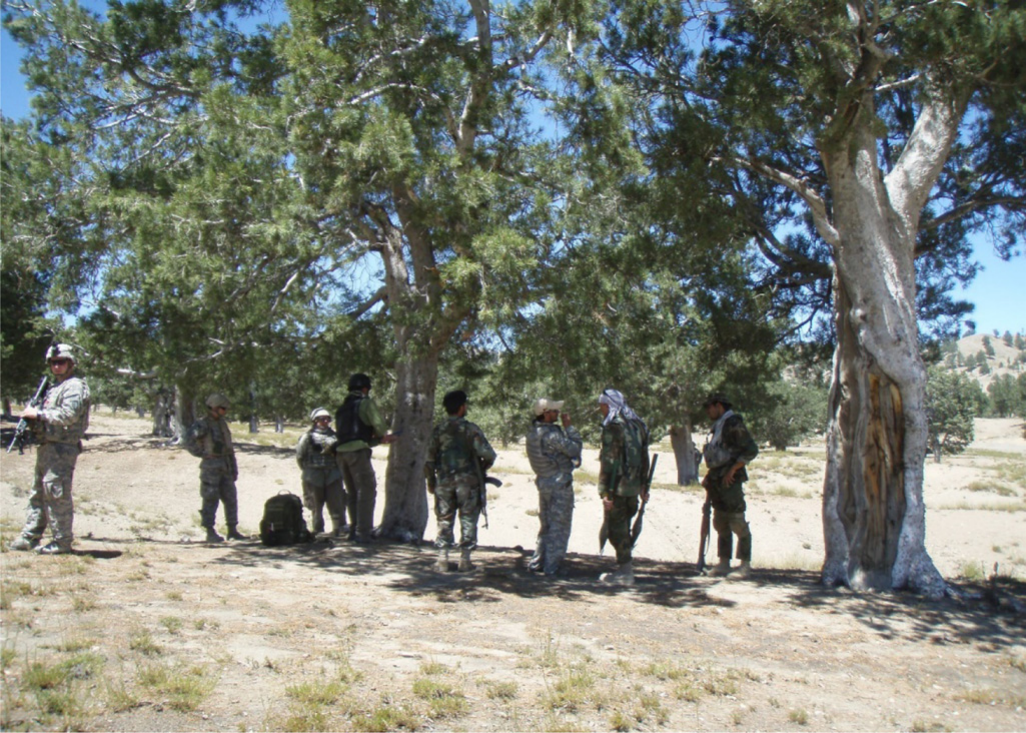
Forest researcher from the U.S. collecting tree growth increment cores as part of a joint U.S.-Afghan Army maneuver. The stand is a semi-natural orchard consisting entirely of nut-bearing chilgoza pine (Sarobi, Paktika Province).

The images (Fig. 8, Fig. 9, Fig. 10, Fig. 11) provide a general view of chilgoza pine forest structure and signs of wood removal in the eastern forest complex of Afghanistan. Fig. 12, Fig. 13 illustrate site measurements accomplished by local surveyor and international scientists with the help of military personnel.

## Footnotes

Transparency document Transparency document associated with this article can be found in the online version at doi:10.1016/j.dib.2018.03.118.
