# Supplementary material for: Data on ecological associations and stand structure of chilgoza pine (Pinus gerardiana Wall. ex D. Don) in Afghanistan
Source: Data Brief. 2018 Mar 30;18:939–46. doi: 10.1016/j.dib.2018.03.118 (PMC5996500; doi:10.1016/j.dib.2018.03.118)
Supplement: Supplementary file 1 — Supplementary material [file mmc1.docx]

There are no conflicts of interest in the preparation or presentation of this manuscript. If there is an additional form that may be signed please contact the corresponding author.

John Groninger
